# Socioeconomic inequalities in the HIV testing during antenatal care: evidence from Indian demographic health survey, 2015–16

**DOI:** 10.1186/s12889-022-13392-6

**Published:** 2022-05-15

**Authors:** Santosh Kumar Sharma, Deepanjali Vishwakarma

**Affiliations:** International Institute for Population Sciences, Govandi Station Road, Deonar, Mumbai, 400088 India

**Keywords:** HIV testing, Antenatal care, Women, Economic inequality

## Abstract

**Background:**

In India, there is currently a lack of data on socioeconomic inequalities in HIV testing on a national scale; thus, understanding socioeconomic inequalities in response to expanded HIV testing is critical for assessing and ensuring equity of HIV programmes in accordance with the Sustainable Development Goals. The specific objective of the study was to determine the factor associated with HIV testing during antenatal care and assess the socio-economic inequalities in HIV testing during antenatal care (ANC) among Indian women aged 15–49 years with a live birth in the two years preceding the survey.

**Methods:**

The results drawn from the state module of women data file of fourth round of National Family Health Survey (NFHS-4, 2015–16), considering HIV testing during antenatal care among women aged 15–49 who gave live birth in the two years preceding the survey and received the result of HIV test as a matter of fact. Method, such as, descriptive statistics, binary ogistic regression, concentration index were used in the analysis.

**Results:**

The findings of the study show that HIV testing during antenatal care was low (30%) among women in India. Our findings reveal that there were significant inequalities exist in HIV testing during ANC between richer and poorer quintile of women. Education, place of residence, comprehensive knowledge of HIV/AIDS, and regular exposure of mass media were substantially contributing to socioeconomic inequality in HIV testing during ANC among women in India.

**Conclusions:**

The socioeconomic inequities in HIV testing during pregnancy should be monitored and addressed in order to ensure an equitable distribution of the benefits specially among children and accomplishments of HIV programs in India.

## Background

Over the last two decades, progress in HIV/AIDS prevention and control have raised concerns about the plausibility of eradicating HIV/AIDS as a public health hazard. The Joint United Nations Programme on HIV/AIDS (UNAIDS) has widely promoted the slogan and goal of eliminating HIV/AIDS by 2030 [1]. According to UNAIDS study, an estimated 5.8 million HIV-positive people lived in the Asia-Pacific area in 2019 [2]. During the 2010–2019 period, the Asia-Pacific region has seen a 12% decline in new HIV infections among total population and 18% among women and girls [2]. Prevention of mother-to-child transmission (PMTCT) has been significantly scaled up across Asia and the Pacific [3]. The period between 2010 and 2017 saw a decline of 33% in new HIV infections among children [4]. HIV prevention programs, such as prevention of parent-to-child transmission (PPTCT), have been identified as an important component in lowering new HIV infections. PPTCT dramatically lowered the risk of HIV infection among newborn children [5–7]). During 2010–18, around 1.4 million children worldwide were prevented from HIV infection with the help of PPTCT [8].

The PMTCT has significantly reduced the risk of HIV infection among newborn children [3, 9]. Prevention of mother-to-child programs to provide a variety of services to women of reproductive ages who are living with or at risk of HIV in order to preserve their health and prevent their newborns from contracting the virus. At various levels of the health care system, PMTCT services are frequently integrated with antenatal and obstetric care [10, 11]. Expansion of HIV testing facilities, particularly for key populations such as pregnant women, adolescents, and HIV-exposed children, is critical to meeting the fast-track targets. Despite attempts to decentralize testing services and enhance community-based testing, coverage remains inadequate. Stigma, prejudice, punitive regulations, and a lack of understanding continue to be significant barriers to testing access [11–13]. A number of studies reported low rates of HIV testing during pregnancy in many countries, despite the progress towards scaling up of the PPTCT program [5, 11, 14, 15]. Several factors were identified that obstacles to HIV testing during pregnancy, such as socio-demographic status, mother’s knowledge of mother-to-child HIV transmission as well as PPTCT, and attitudes towards HIV/AIDS [16–22].

India has the world’s third highest estimated number of HIV/AIDS cases. In 2015, an estimated 2.12 million persons were living with HIV/AIDS, with a child prevalence of 6.54% and an adult prevalence of 0.26% [23]. According to HIV Sentinel surveillance (2014–2015) by the NACO, the overall HIV prevalence is 0.29% among ANC clinic attendees [23]. In India, HIV prevention has been designed with the notion that the key drivers of the pandemic are high-risk individuals, namely commercial sex workers and men who have sex with men, who spread the virus to a male bridge population. This bridge population, primarily migrants and truckers, transmits the virus to their female sexual partners and from them to their offspring. As a result, preventative efforts have focused on high-risk populations and the bridge population in an attempt to break the transmission chain [7, 24]. In India, many barriers prevent the acceptability of Voluntary counseling and HIV testing (VCT). Cultural and social hurdles continue to be disregarded and underappreciated in resource-constrained nations such as India, where VCT clinics have lately been created predominantly in urban areas [25].

Several studies in developing countries have found that the prevalence of HIV testing is lower amongst the poorest and least educated population groups, and it is uncertain whether these inequalities grew or diminished when HIV testing efforts were ramped up [26–30]. Currently, there is a lack of information regarding socioeconomic inequalities in HIV testing on a national scale in India; therefore, understanding socioeconomic inequalities in response to expanded HIV testing is critical to assessing and ensuring equity of HIV programs in accordance with the Sustainable Development Goals. The specific objective of the study is to determine the factor associated with HIV testing during antenatal care and assess the socio-economic inequalities in HIV testing during ANC among Indian women aged 15–49 years with a live birth in the two years preceding the survey.

## Data and methods

### Data

We used unit data from large-scale population-based survey, namely the fourth round of the National Family Health Survey, conducted during 2015–16. NFHS-4 provides compressive information on maternal and child health and HIV-related information across the states and union territories of India. The survey successfully interviewed 601,509 households and 699,686 ever-married women in the age group 15–49, and 112,122 men in the age group of 15–54 across all states and union territories of India. The detailed sampling design, coverage, and findings of the survey are available in the national report [31].

We used the state module of women data file in this study because the HIV section was only covered in this section. The state module collected the information from 122, 351 women. In this study, we gathered information from 122,351 women aged 15–49 at the time of interview about ever been tested for HIV. Then we focused on HIV testing during ANC, which was one of the reported indicators in the HIV testing section of the NFHS-4. Total 17,192 women with a birth two years preceding the survey received HIV test during antenatal care or labour and received results.

## Variables of the study

### Outcome variable

The outcome variable was the HIV testing during antenatal care among women aged 15–49 who gave live birth in the two years preceding the survey and received the result of HIV test (a binary outcome variable coded as yes or no). According to Demographic Health Survey (DHS), HIV testing during antenatal care was defined as number of women who received an HIV test during ANC or labor and received the result of the HIV test with a live birth in the two years preceding the survey [32].

### Independent variable

A set of independent variables were used in the analysis. Selected independent variables included in the study are age group (15–19, 20–24, 25–29, 30–34, 35–39, ≥40), years of schooling (no education, 1–5, 6–9, ≥10), place of residence (urban, rural), religion (Hindu, Muslim, Others (Christian, Sikhs, Jain, etc.)), social caste group (Scheduled Caste [SC], Scheduled Tribe [ST], Other Backward Caste [OBC], Other), Regular exposure of mass media (no, yes), household wealth quintile (poorest, poorer, middle, richer, richest), comprehensive knowledge of HIV/AIDS (no, yes), and region of residence (North, East, Central, North-East, West, South).

The Indian government created a caste classification scheme in contemporary India, dividing the untouchable castes into scheduled castes (SC), backward tribes into scheduled tribes (ST), and disadvantaged castes into other backward castes (OBC). The Forward caste (FC) community is considered to be a high caste. The household wealth index was used as a proxy for household economic status. Economic proxies such as household durable assets, access to good drinking water and sanitation, and landholding were used to calculate the wealth index. The index was created by separating the data into sets of dichotomous variables and then assigning indicator weights using principal component analysis (PCA). The resulting wealth index was split into five quintiles: poorest, poorer, middle, richer, and richest [33].

## Methods

 Descriptive statistics and bivariate analysis were used to explain the women’s characteristics who were tested for HIV during antenatal care. Binary logistic regression was used to determine the factor associated with HIV testing during antenatal care among women. The basic form of the logistic regression model, which yields the probability of occurring of an event, can be depicted as:

$${\mathit{\log}}_e\ \left[P\left({Y}_i=1|{X}_i\right)/1-P\left({Y}_i=1|\ {X}_i\right)\right]={\mathit{\log}}_e\left[\pi |1-\pi \right]=\alpha +{\beta}_1{X}_{i1},\dots \dots \dots ..{\beta}_k{X}_{ik}$$

Where *Yi* is the binary response variable and *X*_*i*_ is the set of explanatory variables such as socio-demographic characteristics, and *β*_*1,*_ *β*_*2,…*_ *β*_*k*_ are the coefficients of the *Xi* variables.

### Concentration index

The concentration index (CI) was used to understand the economic inequalities in HIV testing during antenatal care among women. The concentration indices were used to measure the overall inequalities in HIV testing during antenatal care among the wealth quintiles of women. The concentration index is defined as twice the area between the concentration curve and the line of equality (the 45-degree line) and the index is bounded between − 1 and 1. So, in this case, if there is no socioeconomic-related inequality, the concentration index is zero. The convention is that the index takes a negative value when the curve lies above the line of equality, indicating the disproportionate concentration of the health variable among the poor, and a positive value when it lies below the line of equality. If the health variable is “bad” such as ill health, a negative value of the concentration index means ill-health is higher among the poor.

Formally, the concentration index is defined as.

1 $$C=\frac{2}{\mu}\operatorname{cov}\left({y}_i,{R}_i\right)$$

Where μ is the mean of the outcome variable of the sample and *cov* denotes the covariance; y_*i*_ is the health outcome of the individual, and R is the rank of the individual in the wealth distribution. The sign of the concentration index indicates the direction of any relationship between the health variable and position in the living standards distribution, and its magnitude reflects both the strength of the relationship and the degree of variability in the health variable [34].

## Decomposition of concentration index

For decomposing the concentration index, we have used Wagstaff decomposition analysis. Wagstaff’s decomposition demonstrated that the concentration index could be decomposed into the contributions of each factor to the income-related inequalities. Each contribution is the outcome of the sensitivity of health concerning that socio-economic factor and the extent of income-related inequality in that factor. Based on the linear regression relationship between the outcome variable *Y*_*i*_, the intercept α, the relative contribution of X_*ki*_ and the residual error *ε*_*i*_ in the Eq. 2,

2 $${Y}_i=\alpha +\sum {\beta}_k{X}_{ki}+\kern0.5em {\varepsilon}_i$$

where *ε*_*i*_ is an error term, given the relationship between *Y*_*i*_, and *X*_*ki*_ in the above equation, and CI for *Y*_*i*_ can be rewritten as in below equation:

3 $$C=\sum \frac{\beta_k{\overline{X}}_k}{\mu}\kern0.5em {C}_k+\kern0.5em \frac{GC_{\varepsilon }}{\mu }$$

where μ is the mean of *Y*_*i*_*,* ${\overline{X}}_k$ is the mean of *X*_*k*_*, β*_*k*_ is the coefficient from a linear regression of outcome variables, *C*_*k*_ is the concentration index for *X*_*k*_ (defined analogously to C, and *GCε* is the generalized concentration index for the error term (*ε*_*i*_). Equation (3) shows that C is the outcome of two components: First, the determinants or ‘explained’ factors, which are equivalent to the weighted accumulation of the concentration indices of the regressor, where one unit change in the outcome variable is to be associated with the one unit change in the explanatory variable. Second, a residual or ‘unexplained’ factor indicating the inequality in health variable that cannot be explained by selected explanatory factors across various socioeconomic groups [35].

All the statistical analyses were conducted by Stata®16 (StataCorp LLC, Lakeway Drive College Station, Texas, USA), using the weighted women-related variables in the dataset.

## Results

Table 1 presents the prevalence of HIV testing during antenatal visits or labour and received results among women with a birth 2 years before the survey in India according to some selected background characteristics. It was observed that all the background characteristics included in the study were significantly (*p* < 0.001) associated with HIV testing during antenatal or labour and received results among women with a birth 2 years before the survey in India. Overall, 29.8% (95% CI: 29.1, 30.5) of women received HIV test during ANC visit or labour and received results among women with a birth 2 years before the survey. HIV testing during ANC visit increased significantly with years of schooling, from 7.5% (95% CI: 6.7, 8.3) in the no education group to 51.8% (95% CI: 50.5, 53.0] in the ten and above year of schooling group during ANC. The prevalence of HIV testing during ANC was higher in urban areas [46.1% (95% CI: 44.7,47.5)] compared to rural areas [23.1% (95% CI: 22.3, 23.9)]. It was found that women belonging to other religious groups (e.g., Christian, Sikhs, Jain, etc.) [51.7% (95% CI: 48.1, 55.2)] reported a higher prevalence of HIV testing during ANC than the Hindu [29.5% (95% CI: 28.7, 30.3)] and Muslim [25.0% (95% CI: 23.4, 26.7)]. Similarly, the prevalence of HIV testing during ANC was higher among women belonging Others social caste group [34.3% (95% CI: 32.9, 35.8)]. It was found that the prevalence of HIV testing during ANC was higher among women who had regular exposure to media [40.3% (95% CI: 39.4, 41.2)] and had comprehensive knowledge of HIV/AIDS [53.5% (95% CI: 51.6, 55.4)]. The prevalence of HIV testing during ANC among women aged 15–49 increased with better economic status, from 6.9% (95% CI: 6.2, 7.8) in the poorest to 52.5% (95% CI: 51.6, 55.4) in the richest quintile. Regional variation in HIV testing was also observed among women. The prevalence of HIV testing was highest in the Southern region of India [70.1% (95% CI: 68.6, 71.7)], followed by Western [40.7% (95% CI: 38.7, 42.6)], Northern [29.1% (95% CI: 27.2, 31.0)], North-eastern [16.6% (95% CI: 13.7, 20.0)], Eastern [12.0% (95% CI: 11.1, 13.1)], and Central region (11%).

**Table 1:** Percentage of women received HIV test during ANC visit or labour and received results among women with a birth 2 years before the survey in India, 2015–16

Background Characteristics	Number of women	% of women received HIV test during ANC visit or labor and received results among women with a birth 2 years before the survey [95% CI]	Chi square
			[***p*** value]
**Age (Years)**			
15–19	971	28.7 [25.8–31.6]	72.6
20–24	7080	31.1 [30.0–32.2]	[*P* = 0.000]
25–29	5854	30.6 [29.4–31.8]	
30–34	2351	28.9 [27.1–30.8]	
35–39	728	22.2 [19.3–25.4]	
40 and above	208	5.7 [3.2–9.9]	
**Years of schooling**			
No education	4498	7.5 [6.7–8.3]	2300
1–5	2115	16.8 [15.3–18.5]	[*P* = 0.000]
6–9	4417	28.1 [26.8–29.5]	
10 and above	6162	51.8 [50.5–53.0]	
**Residence**			
Urban	5023	46.1 [44.7–47.5]	619.5
Rural	12,169	23.1 [22.3–23.9]	[*P* = 0.000]
**Religion**			
Hindu	13,519	29.5 [28.7–30.3]	264.4
Muslim	2871	25.0 [23.4–26.7]	[*P* = 0.000]
Others	802	51.7 [48.1–55.2]	
**Caste**			
SC/ST	5373	25.9 [24.7–27.1]	145.5
OBC	7697	30.1 [29.1–31.2]	[*P* = 0.000]
Others	4122	34.3 [32.9–35.8]	
**Regular exposure of media**			
No	5673	8.4 [7.7–9.2]	1700.2
Yes	11,519	40.3 [39.4–41.2]	[*P* = 0.000]
**Wealth index**			
Poorest	4026	6.9 [6.2–7.8]	2400.6
Poorer	3633	17.7 [16.5–19.0]	[*P* = 0.000]
Middle	3588	32.9 [31.4–34.5]	
Richer	3143	48.4 [46.6–50.1]	
Richest	2802	53.5 [51.6–55.4]	
**Comprehensive knowledge about HIV/AIDS**			
No	13,629	23.5 [22.8–24.2]	1200.9
Yes	3563	54.0 [52.4–55.7]	[*P* = 0.000]
**Region**			
North	2269	29.1 [27.2–31.0]	2600.4
Central	4284	10.8 [9.8–11.7]	[*P* = 0.000]
East	4133	12.0 [11.1–13.1]	
Northeast	570	16.6 [13.7–20.0]	
West	2555	40.7 [38.7–42.6]	
South	3381	70.1 68.6–71.7]	
**Total**	17,192	29.8 [29.1–30.5]	

*CI* confidence intervals

Table 2 presents the results of adjusted odds ratio (AOR) of HIV testing during ANC visit or labor and received results among women aged 15–49 with a birth two years before the survey in India. The tables showed that there are gradient effect of socio-dmeogtraphic and economic factors on HIV testing during ANC visit or labor and received results among women aged 15–49 with a birth two years before the survey. The likelihood of HIV testing during ANC was significantly higher among women aged 20–39 years than the 15–19 years of women. The Odds of HIV testing during ANC increased with years of schooling from 1.5 times (*p* < 0.001) in the no education group to 3.4 times (*p* < 0.001) in the 10+ year of schooling group. Women residing in rural areas were significantly less likely [AOR = 0.84; 95% CI: 0.77, 0.93] to be tested for HIV during ANC than the urban areas. Women belonging to other religious groups (e.g., Christian, Sikhs, Jain, etc.) had significantly higher odds of HIV testing during ANC [AOR = 1.67; 95% CI: 1.44, 1.92] than their other counterparts. Women having regular exposure of mass media [AOR = 1.91; 95% CI: 1.69, 2.16] and comprehensive knowledge of HIV/AIDS [AOR = 2.39; 95% CI: 2.18, 2.62] had significantly higher odds of HIV testing during ANC.

**Table 2:** Binary logistic regression of HIV testing during ANC visit or labour and received results among women aged 15–49 with a birth 2 years before the survey in India, 2015–16

	Adjusted Odds ratio (AOR)	95% CI
**Age (Years)**		
15–19®		
20–24	1.302***	(1.06, 1.59)
25–29	1.494***	(1.22, 1.83)
30–34	1.607***	(1.29, 1.99)
35–39	1.620***	(1.24, 2.12)
40 and above	0.975	(0.62, 1.53)
**Years of schooling**		
No education®		
1–5	1.492***	(1.26, 1.77)
6–9	2.257***	(1.96, 2.60)
10 and above	3.370***	(2.91, 3.9)
**Residence**		
Urban®		
Rural	0.844***	(0.77, 0.93)
**Religion**		
Hindu®		
Muslim	1.058	(0.94, 1.19)
Others	1.667***	(1.44, 1.92)
**Caste**		
SC/ST®		
OBC	0.805***	(0.72, 0.89)
Others	1.057	(0.94,1.19)
**Regular exposure of media**		
No®		
Yes	1.907***	(1.69, 2.16)
**Wealth index**		
Poorest®	1.343***	(1.14, 1.58)
Poorer	1.654***	(1.39, 1.96)
Middle	2.137***	(1.78, 2.57)
Richer	2.491***	(2.04, 3.04)
Richest		
**Comprehensive knowledge about HIV/AIDS**		
No®		
Yes	2.388***	(2.18, 2.62)
**Region**		
North®		
Central	0.436***	(0.38, 0.50)
East	0.691***	(0.60, 0.80)
Northeast	1.140*	(0.98, 1.32)
West	1.319***	(1.14, 1.52)
South	4.641***	(4.04, 5.33)
Constant	0.040	(0.03, 0.05)

® Reference; * *p* < 0.10; ** *p* < 0.05; *** *p* < 0.01; *CI* confidence intervals; Adjusted odds ratios were estimated after adjusting the socio-demographic and socioeconomic variables

The likelihood of HIV testing during ANC was higher among women belonging to the better economic status of households. It was found that as the wealth quintile increased, the likelihood of HIV testing during ANC also increased in India. For instance, the odds of HIV testing during ANC increased from 1.3 times (*p* < 0.001) in the poorest quintile to 2.5times (*p* < 0.001) in the richest quintile. The odds of HIV testing during ANC were higher in the southern region [AOR = 4.64; 95% CI: 4.04, 5.33] of India than the other counterparts.

**Fig. 1:**
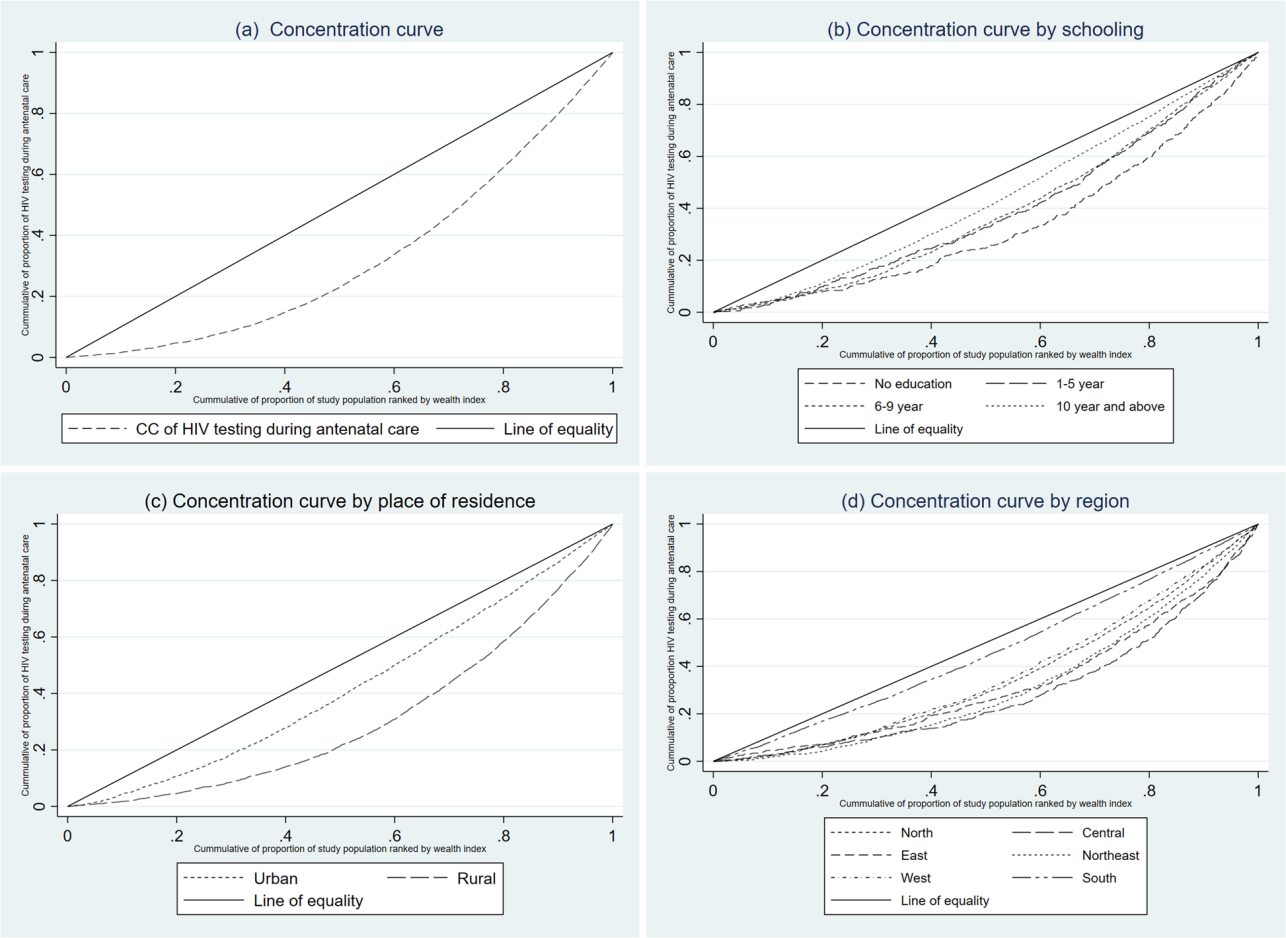
Concentration curve for HIV testing during ANC visit or labour and received results among women aged 15–49 with a birth 2 years before the survey in India, 2015–16

Table 3 presents the concentration indices (CI) of HIV testing during ANC visit or labor and received results among women aged 15–49 with a birth two years before the survey in India according to some selected background characteristics. Economic inequalities with respect to HIV testing during ANC among women aged 15–49 refer to the degree to which HIV testing during ANC rates differs between less or more economically advantageous groups. In this study, economic inequality with respect to HIV testing during ANC was measured using concentration index and concentration curve. Overall, Table 3 and Fig. 1a shows that HIV testing during ANC concentrated more in affluent class of women, as shown in Fig. 1a, concentration curve below the equity line. The overall value of concentration index was 0.334. It was found that inequality in HIV testing during ANC between the poor and the rich was higher among women aged 35 and above.

**Table 3:** Concentration indices of HIV testing during ANC visit or labour and received results among women aged 15–49 with a birth 2 years before the survey in India, 2015–16

	Concentration index	Standard error	***p***-value
**Age (Years)**			
15–19	0.458	0.041	0.000
20–24	0.444	0.014	0.000
25–29	0.480	0.015	0.000
30–34	0.581	0.022	0.000
35–39	0.642	0.041	0.000
40 and above	0.835	0.144	0.000
**Years of schooling**			
No education	0.410	0.031	0.000
1–5	0.306	0.032	0.000
6–9	0.312	0.018	0.000
10 and above	0.217	0.015	0.000
**Residence**			
Urban	0.264	0.017	0.000
Rural	0.491	0.011	0.000
**Religion**			
Hindu	0.473	0.011	0.000
Muslim	0.587	0.022	0.000
Others	0.428	0.024	0.000
**Caste**			
SC/ST	0.488	0.015	0.000
OBC	0.499	0.014	0.000
Others	0.461	0.018	0.000
**Regular exposure of media**			
No	0.416	0.026	0.000
Yes	0.307	0.011	0.000
**Comprehensive knowledge about HIV/AIDS**			
No	0.486	0.011	0.000
Yes	0.284	0.019	0.000
**Region**			
North	0.467	0.020	0.000
Central	0.518	0.027	0.000
East	0.399	0.030	0.000
Northeast	0.540	0.031	0.000
West	0.335	0.029	0.000
South	0.219	0.028	0.000
Total	0.334	0.004	0.000

Table 3 and Fig. 1b shows that as the level of education increases, concentration curve tending towards the equality line. It means inequality in HIV testing during ANC consistently decreased as the level of education increased. Similarly, concentration curve by place of residence shows that inequality in HIV testing during ANC was higher in rural areas (CI: 0.49; SE: 0.01) compared to urban areas (CI: 0.264; SE: 0.02) (Fig. 1c). The value of concentration index indicates that the economic inequality in HIV testing was lower among women having regular exposure of mass media (CI: 0.31; SE: 0.01) and comprehensive knowledge of HIV/AIDS (CI: 0.28; SE:0.02). In the case of religion, the inequality in HIV testing during ANC was significantly higher in Muslim religion as compared to Hindu and another religious group. The inequality in HIV testing during ANC was highest in Northeast (CI:0.54; SE: 0.03) followed by Central (CI: 0.52; SE:0.03), North (CI: 0.47; SE: 0.02), East (CI: 0.40; SE:0.03), West (CI: 0.33; SE:0.03), and South (CI: 0.22; SE:0.03) region of India (Fig. 1d).

Table 4 presents the decomposition of concentration index of HIV testing during ANC visit or labour and received results among women aged 15–49 with a birth 2 years before the survey in India. Decomposition results shows that the largest contribution to inequality in HIV testing during antenatal care was attributable to women’s education (29%), i.e., if education were equally distributed among women having different level of education, then inequality in HIV testing during antenatal care would decline by 26%. Similarly, place of residence, regular exposure of mass media, comprehensive knowledge of HIV/AIDS made a substantial contribution to observed inequality in HIV testing during ANC, explaining 9, 8, and 10% of the total inequality respectively. It was found that southern region (18%) showed a significant contribution to observed inequality in HIV testing during ANC than the other region.

**Table 4:** Estimates of Marginal effect and decomposition analysis for contribution of explanatory factors to the inequality in HIV testing during ANC visit or labour and received results among women aged 15–49 with a birth 2 years before the survey in India, 2015–16

	Elasticity	CI	Absolute Contribution to CI	% Contribution to CI
**Age (Years)**				
15–19				
20–24	0.007	0.005	0.000	0.0
25–29	0.012	0.003	0.000	0.0
30–34	0.015	0.009	0.000	0.0
35–39	0.019	0.008	0.000	0.1
40 and above	−0.016	0.045	−0.001	−0.2
**Years of schooling**				
No education				
1–5	0.007	−0.162	−0.001	−0.4
6–9	0.073	−0.030	−0.002	−0.7
10 and above	0.254	0.343	0.087	26.0
**Residence**				
Urban				
Rural	−0.132	−0.228	0.030	9.0
**Religion**				
Hindu				
Muslim	0.003	0.031	0.000	0.0
Others	0.022	0.221	0.005	1.5
**Caste**				
SC/ST				
OBC	−0.034	0.018	− 0.001	− 0.2
Others	0.002	0.223	0.001	0.1
**Regular exposure of media**				
No				
Yes	0.159	0.173	0.028	8.3
**Comprehensive knowledge about HIV/AIDS**				
No				
Yes	0.116	0.288	0.033	10.0
**Region**				
North				
Central	−0.080	−0.166	0.013	4.0
East	− 0.056	− 0.345	0.019	5.8
Northeast	−0.010	−0.222	0.002	0.7
West	0.055	0.181	0.010	3.0
South	0.298	0.200	0.060	17.8
Explained CI			0.283	100
Total CI			0.334	
Residual			0.059	

## Discussion

Offering HIV testing during ANC as a part of PMTCT has resulted in significant reductions in new paediatric HIV infections and expanded ART coverage for women in many countries. Receiving HIV testing services as early as possible during pregnancy ensures HIV-positive pregnant women to get the most out of prevention, medication, and care, as well as reduce the chance of HIV infection to their children [36]. This is the first study that attempted to assess the socioeconomic correlates of HIV testing during antenatal care among women aged 15–49 who had a birth two years prior to the survey using NFHS-4 data. For the first time, the NFHS-4 gathered data on HIV testing during antenatal care among Indian women. The specific objective of this study was to determine the factor associated with HIV testing during ANC and to measure the socioeconomic inequalities in HIV testing during antenatal care. The following are the salient findings of the study.

Since 2016, over 21 thousand ICTCs have been available across India to provide free services to pregnant women, with the majority of them being linked to government-funded healthcare institutions [37]. Despite the implementation of PPTCT program of the NACO that offers routine HIV testing to all pregnant mothers [38], only 30% of mothers experienced HIV testing during ANC in India resultant from our study. This prevalence is higher than the prevalence of HIV testing among all women aged 15–49 reported in previous study using NFHS-4 data [39]. Besides, comparing with other studies conducted in developing countries using the DHS data and using the same definition, the prevalence of HIV testing did not differ much from theirs [5, 14].

Several studies have documented that there is a significant association between sociodemographic characteristics and HIV testing during pregnancy among women [14, 17, 19, 40]. We found that HIV testing during antenatal care increases with increasing level of education. Our findings are consistent with previous studies conducted in many developing countries such as Asian and African countries [5, 14, 41–43]. It may be due to the reason that education might enhance HIV-related awareness and earnings among women, which leads to increased use of maternal health care services [19, 44]. Furthermore, educated women may be more exposed to HIV/AIDS-related facts, have a better understanding of the benefits of HIV treatment, and be better able to make informed decisions about whether or not to get tested [45]. Regular exposure of mass media and comprehensive knowledge of HIV/AIDS played as an important catalyst to have HIV testing during ANC among mothers. This finding was coupled with the other studies conducted in developing countries [19, 46, 47]. The findings of the study also revealed a large gap in the uptake of HIV testing during ANC between urban (46%) and rural areas (23%), as the HIV testing prevalence was almost twice in urban areas than the rural. It might be due to the fact that more than 70% of India’s population resides in rural areas, as a result large number of HIV positive women will be left undiagnosed if rural women have no access to HIV testing [11, 48]. We found that the likelihood of HIV testing during antenatal care was higher in southern region of India. It may be due to the fact that most of the HIV related program were mainly focused in high HIV prevalent states of India [23, 49].

Findings of the study revealed that as the wealth index increased from the lowest to the wealthiest quintile, the likelihood of HIV testing during ANC rose. In particular, socioeconomic status was substantially correlated with women’s attendance at HIV testing. This finding revealed that poverty is significant influencing factor associated with HIV testing during ANC. Our findings, which showed that there is significant inequality in HIV testing during ANC between richer and poorer quintile of women, are consistent with findings from low -middle-income countries [20, 50, 51]. We found that education, place of residence, regular exposure of mass media, and comprehensive knowledge of HIV/AIDS made a considerable contribution to observed socio-economic inequalities in HIV testing during antenatal period. It’s possible that demand for HIV testing is lower in the poorest and less educated sectors of the population because people in these groups believe they are less vulnerable to AIDS [52].

There were few limitations in our study. First, the study’s findings were based on self-reported outcomes. Recall and reporting bias might affect the self-reported information. The validity of self-reported HIV testing is difficult to assess, especially because accuracy varies depending on HIV status. Because inequality assessments rely on quantifying a relationship, differences in self-reporting accuracy between socioeconomic groups might have skewed our findings [19, 53]. Second, the NFHS data did not allow users to determine if a mother refused or accepted an HIV test after being offered one during ANC, which might be associated to socioeconomic status. Furthermore, despite the fact that HIV testing was readily available as part of ANC, many Indian women were only tested for the first time when they went into labour, and so did not receive the full advantage of the PPTCT programme. Despite this, the study’s findings are based on large-scale national survey data, which has the ability to provide insight for policymakers and programme planners in developing suitable national and regional intervention strategies.

## Conclusion

The findings of the study conclude that HIV testing during antenatal care was low among women in India. Several sociodemographic and economic characteristics were significantly associated with HIV testing during antenatal care. Education, place of residence, comprehensive knowledge of HIV/AIDS, and regular exposure of mass media were substantially contributing to socioeconomic inequality in HIV testing during ANC among women in India. HIV policies and program are not likely to reach every segment of the community without a strong focus on equality, especially unreachable to poorest an least educated section of population. The socioeconomic discrepancies in HIV testing during pregnancy should be monitored and addressed in order to ensure an equitable distribution of the benefits and accomplishments of HIV programs in India. The findings emphasize the need for a comprehensive strategy to HIV testing for pregnant women who are at a higher risk of not receiving it. Because not all women are offered an HIV test, further study is needed to find the barriers that remain undiscovered.

## Data Availability

All result-based data is included in the manuscript, and the data sets are available online at www.measuredhs.com for anybody to access.

## Abbreviations

HIV: Human Immunodeficiency Virus
AIDS: Aquired Immunodeficieny Syndrome
ANC: Anetnatal care
DHS: Demographic Health Survey
CI: Concentration index
PPTCT: Prevention of parent-to-child transmission
PMTCT: Prevention of mother-to-child transmission of HIV
SE: Standard error
